# Adapting DIALOG+ in a School Setting—A Tool to Support Well-being and Resilience in Adolescents Living in Postconflict Areas During the COVID-19 Pandemic: Protocol for a Cluster Randomized Exploratory Study

**DOI:** 10.2196/40286

**Published:** 2022-11-09

**Authors:** Carlos Gómez-Restrepo, María José Sarmiento-Suárez, Magda Alba-Saavedra, Victoria Jane Bird, Stefan Priebe, Francois van Loggerenberg

**Affiliations:** 1 Departamento de Epidemiologia Clínica y Bioestadistica Departamento de Psiquiatría y Salud Mental Pontificia Universidad Javeriana - Hospital Universitario San Ignacio Bogotá Colombia; 2 Facultad de Medicina Pontificia Universidad Javeriana Bogotá Colombia; 3 The Unit for Social and Community Psychiatry Wolfson Institute of Population Health Queen Mary University of London London United Kingdom; 4 Youth Resilience Unit Wolfson Institute of Population Health Queen Mary University of London London United Kingdom

**Keywords:** mental health, mental disorder, eHealth, digital health, digital intervention, psychosocial intervention, resilience, psychological support, psychosocial well-being, mental well-being, resource-oriented approach, computer-mediated intervention, armed conflict, post-conflict, adolescent health, adolescent, adolescence, child, youth, school, teacher, student, acceptability, feasibility, vulnerable

## Abstract

**Background:**

Colombia has a long history of an armed conflict that has severely affected communities with forced internal displacement and violence. Victims of violence and armed conflicts have higher rates of mental health disorders, and children and adolescents are particularly affected. However, the mental health needs of this population are often overlooked, especially in low- and middle-Income countries, where scarcity of resources exacerbates the problem that has been further compounded by the global COVID-19 pandemic. Thus, special attention should be paid to the development of interventions that target this population.

**Objective:**

Our research aims to adapt an existing patient-centered digital intervention called DIALOG+ from a clinical setting to an educational setting using stakeholders’ (teachers’ and students’) perspectives. We aim to evaluate the feasibility, acceptability, and estimated effect of implementing this intervention as a tool for the identification and mobilization of personal and social resources to mitigate the impact of social difficulties and to promote mental well-being.

**Methods:**

We will conduct an exploratory mixed methods study in public schools of postconflict areas in Tolima, Colombia. The study consists of 3 phases: adaptation, exploration, and consolidation of the DIALOG+ tool. The adaptation phase will identify possible changes that the intervention requires on the basis of data from focus groups with teachers and students. The exploration phase will be an exploratory cluster randomized trial with teachers and school counselors to assess the acceptability, feasibility, and estimated effect of DIALOG+ for adolescents in school settings. Adolescents’ data about mental health symptoms and wellness will be collected before and after DIALOG+ implementation. During this phase, teachers or counselors who were part of the intervention group will share their opinions through the think-aloud method. Lastly, the consolidation phase will consist of 2 focus groups with teachers and students to discuss their experiences and to understand acceptability.

**Results:**

Study recruitment was completed in March 2022, and follow-up is anticipated to last through November 2022.

**Conclusions:**

This exploratory study will evaluate the acceptability, feasibility, and estimated effect of DIALOG+ for adolescents in postconflict school settings in Colombia. The use of this technology-supported tool aims to support interactions between teachers or counselors and students and to provide an effective student-centered communication guide. This is an innovative approach in both the school and the postconflict contexts that could help improve the mental health and wellness of adolescents in vulnerable zones in Colombia. Subsequent studies will be needed to evaluate the effectiveness of DIALOG+ in an educational context as a viable option to reduce the gap and inequities of mental health care access.

**Trial Registration:**

ISRCTN Registry ISRCTN14396374; https://www.isrctn.com/ISRCTN14396374?q=ISRCTN14396374

**International Registered Report Identifier (IRRID):**

DERR1-10.2196/40286

## Introduction

Colombia has traditionally been considered a violent country as a consequence of a long internal armed conflict, which has caused high rates of internal displacement and violence [1]. The number of individuals affected by this conflict has been estimated to approach 9.2 million [2], and despite the peace agreements signed in 2016, the problem persists in many regions of the country [3]. Worryingly, it is estimated that approximately 700,000 people have been affected by internal displacement ever since these agreements were reached [4].

One of the most affected regions in Colombia is the department of Tolima. Its central-western location in the country has made it a strategic corridor for trafficking of narcotics and the armed conflict [5]. Within Tolima, certain rural municipalities including Chaparral, Rioblanco, and Planadas have been particularly affected because of the connection they provide between the Pacific region of Colombia and the center of the country [6]. To prioritize these municipalities, which have been constantly subjected to armed conflict, in 2017, the Colombian government designated territories including Chaparral and Rioblanco as part of a Development Program with a Territorial Approach (PDET). This program aims to accelerate transformation and access to services for the people living in these regions [6,7].

The impact of armed conflict on the people inhabiting these regions is considerable, and international crises and wars have unveiled the consequences that these conditions have on mental health. Several studies demonstrate that individuals displaced by violence are at an increased risk of mental health disorders [3,8,9]. These include posttraumatic stress disorder (PTSD), depression, anxiety, and mood and sleep disorders, among others [10,11]. Even more concerning is the impact that these conflicts have on children and adolescents—a population that tends to have higher susceptibility to mental health conditions [12]. This may be due to the key psychological, biological, and social changes that characterize this period of life [13].

In Colombia, the National Mental Health Survey conducted in 2015, using screening scales, calculated a lifetime prevalence of any mental health disorder of 4.7% in children aged between 7 and 11 years and of 7.2% in adolescents aged 12 to 17 years [14]. Additionally, and in accordance with the worldwide trend, mental health conditions seem to be more prevalent in children and adolescents who have been subjected to internal displacement: the prevalence of an anxiety disorder and PTSD was higher (6.5% and 13.2%, respectively) in children (aged 7 to 11 years) who had experienced displacement due to violence, compared to an estimated prevalence of 1.8% for any anxiety disorder and 6.6% for PTSD in those who had not gone through the same experience [15]. Similarly, the 12- to 17-year-old group who had been displaced had a higher prevalence of any anxiety and depression disorders. Worryingly, the same significant difference in prevalence was reported for suicidal thoughts (19.8%) and suicide attempts (9.1%), which were higher in those who were displaced than in those who were unaffected (5.8% and 2.1%, respectively) [16].

Despite this clear problem and multiple issues, both governmental and individual barriers persist and impede an adequate response to the mental health needs of this vulnerable population [17]. It is estimated that in low- and middle-income countries, there is a significant shortage of mental health professionals such as child psychiatrists, accounting for only 0.1 per 100.000 population [18]. In Colombia, there are 1584 psychiatrists, which implies that there are 3 psychiatrists for every 100,000 inhabitants. However, psychiatrists are concentrated in the capital cities, which prevents access to people in rural areas owing to travel, time, and costs. This is an especially significant barrier to children and adolescents' access to mental health services [19]. Furthermore, the treatment gap (the gap between those requiring and receiving adequate treatment) for mental health disorders in children and adolescents of Central and South America has been estimated to be between 64% and 86%, which is an alarmingly high estimate [17]. According to the Pan American Health Organization, the treatment gap for any mental health disorder in Colombia is 86.1% and was the highest of all the countries in the Americas, followed by Guatemala (84.9%) and México (81.4%) and including the United States (58.9%) [20]. Moreover, the recent COVID-19 pandemic and its restrictions have exacerbated mental distress. Many studies have identified particular stressors that the COVID-19 isolation measures had on children and adolescents: lack of social contact, lack of personal space at home, separation from parents or caregivers, and concerns about academic and social impacts [21,22], all of which can lead to the development of anxiety or depressive disorders [23-25]. As in most countries, lockdown measures have been eased as of this writing, and it is important to keep investigating the impact that these restrictive measures had on children and to consider that the results obtained have to be viewed in a postquarantine context.

The aforementioned barriers, as well as the scarcity of funds allocated to mental health and the lack of education about and community knowledge of mental health issues, can perpetuate the stigma and false beliefs that surround mental health disorders. The lack of education and community knowledge highlights the essential role that teachers and school counselors can play in promoting mental health, well-being, and resilience in children and adolescents [26]. One of the biggest challenges in Colombia relates to the lack of personnel trained in mental health in schools in rural areas where there is usually only 1 counselor per school, who is not always a psychological professional. Interventions that aim to increase and inculcate skills concerning mental health in educators (such as courses or capacity development initiatives that focus on mental health) are fundamental [26,27].

Likewise, the school environment allows a constant interaction among teachers, counselors, and their students, which encourages the creation of a bond based on trust and provides the educators a chance to identify potential mental health disorders [28]. Therefore, an approach to mental health issues in the school setting can aid in overcoming some of the previously mentioned barriers [29].

To promote this approach in schools, we propose implementing an existing patient-centered digital intervention called DIALOG+, which was developed to facilitate the interaction between the clinician and patients with mental health issues [30-33]. This has primarily been implemented in mental health care settings. However, its adaptation outside of the clinical context and into the school context is innovative and has not been attempted before. Through this study, we aim to evaluate the feasibility of implementing DIALOG+ in the educational setting through teachers and counselors as a tool for the identification and mobilization of personal and social resources to mitigate the impact of social difficulties and to enhance quality of life.

## Methods

### DIALOG+

This tool is a resource-oriented, evidence-based intervention developed to facilitate the interaction between the clinician and patients with a mental health condition, allowing patients to actively participate in clinical meetings [34]. By structuring the interaction that takes place during routine clinical meetings, it encourages self-reflection and expression and empowers patients to improve their mental and social situation themselves. The intervention is supported by an app that can be installed on multiple electronic devices (eg, a tablet or cellphone).

During consultation, and with a patient-centered assessment, DIALOG+ invites the individual to evaluate their satisfaction with 8 life domains (mental health, physical health, work, accommodation, leisure activities, friends, relationship with family or partners, and personal safety) and 3 treatment domains (medication, practical help, and professional appointments). Each item is rated on a scale of 1=“totally dissatisfied” to 7=“totally satisfied,” and patients are asked if they would like additional help with that domain.

Scores are summarized and displayed on the app at subsequent meetings, allowing comparisons with previous scores from previous meetings. Physicians provide positive feedback on any domain showing improvement. This is followed by a 4-step, solution-focused approach to identify psychosocial resources to intervene in up to 3 domains, which the patient has identified as needing assistance to improve their quality of life. DIALOG+ is an evidence-based and effective intervention [32] that has shown to save up to £1345 (US $1510.80) per patient for the UK health system, mainly owing to fewer days of hospitalization among individuals receiving the intervention [30,35].

### Intervention and Study Design

#### Overview

This study will adapt the DIALOG+ intervention through a mixed methods study. The quantitative component comprises an exploratory cluster randomized controlled trial. Initially, we will measure variables concerning mental health, resilience, quality of life, and social, and familiar functionality. These will be measured once more after the intervention stage. The qualitative component consists of conducting focus groups with teachers and students, and the think-aloud method [36] to obtain insights into the DIALOG+ adaptation in the school setting.

**Figure 1:**
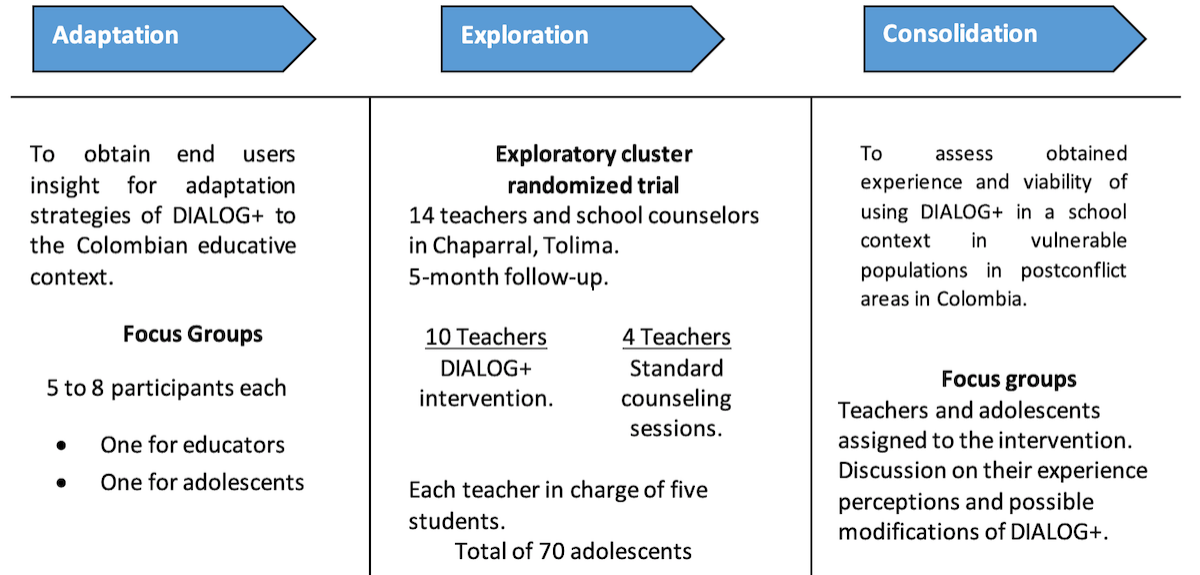
Adaptation, exploration, and consolidation phases.

The study design has 3 phases: adaptation, exploration, and consolidation (Figure 1).

#### Adaptation

We will present the existing DIALOG+ tool to teachers and adolescents between 12 and 18 years of age during focus groups to obtain end users’ insights into adaptation strategies of DIALOG+ for the Colombian educative context. We will conduct one focus group with adolescents and another one with teachers and educators recruited through convenience sampling. Each focus group will include between 5 and 8 participants who will first sign an informed consent or assent form, and they will then be audio recorded for transcription and analysis. The information obtained in this phase will be key for adapting the intervention and for developing the following phase of the study.

#### Exploration

**Figure 2:**
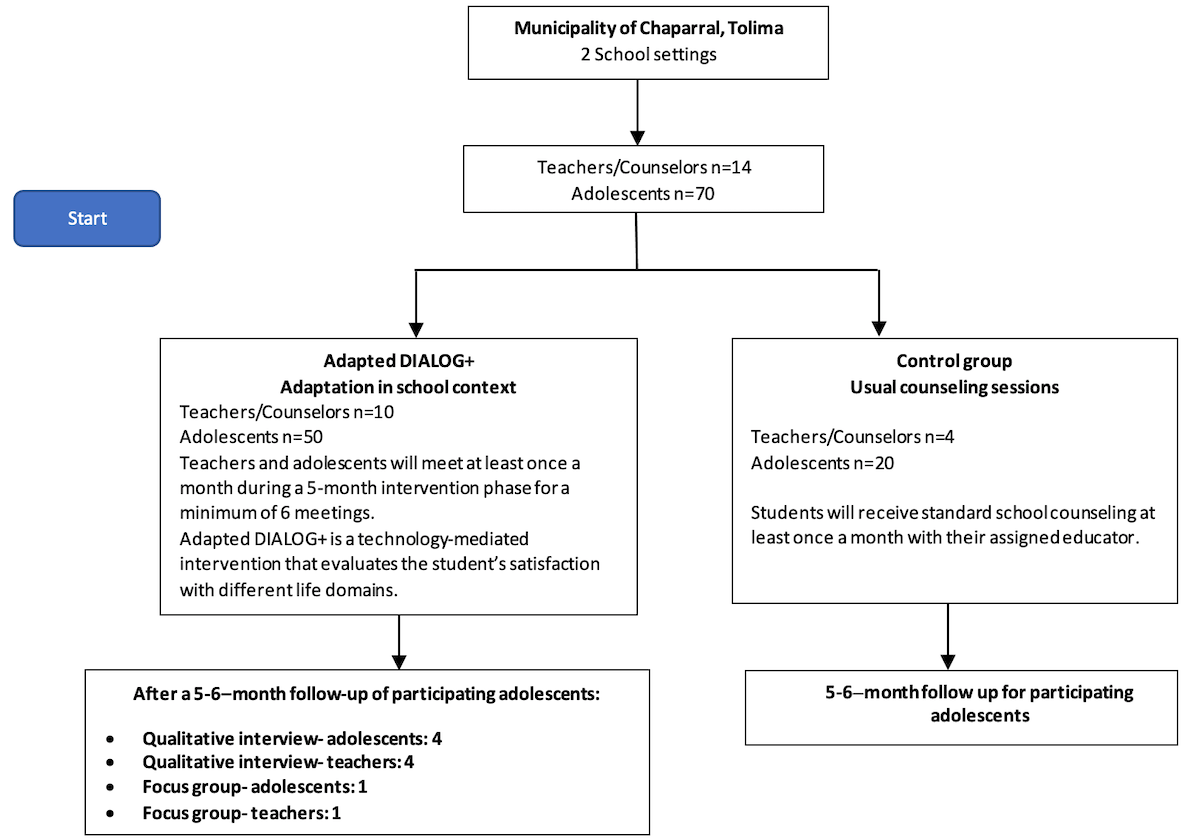
Exploratory cluster randomized trial.

This phase consists of an exploratory cluster randomized trial with 14 teachers and school counselors in Chaparral, Tolima. This specific phase will assess the acceptability, feasibility, and estimated effect of applying the DIALOG+ intervention in school settings. Teachers and their students (who together form a cluster) will be randomly allocated to either the experimental (DIALOG+) group or an active control group (counseling as usual). Teachers will act as the unit of randomization with clustering by teacher to prevent contamination effects within the study. Accordingly, we will have 14 clusters comprising 1 teacher and 5 students each. The unit for randomization will be teachers in a ratio of 10:4 to the intervention and the control groups to maximize our data on feasibility and acceptability of the intervention, while providing some comparison data in the control group to estimate effect. Teachers will be recruited first, and they will then identify eligible students. Each teacher will invite 5 adolescents who they consider to be in need of counseling or additional support for presenting a personal, family, or social situation that is affecting their performance at school or their well-being. Students who accept the invitation will be asked to sign the assent and informed consent of their parents. Participants will be excluded from the study if they intend to change towns or school in the near future, or if they cannot participate for the full duration of the study. The teachers will determine the students with whom they will participate in the study. Teachers and their students (who together form a cluster), will be randomly allocated to either the experimental (DIALOG+) group or to an active control group (counseling as usual) by the study coordinator who is blinded to their identities. Each teacher or counselor will be in charge of 5 students during the intervention for a total of 70 students (Figure 2).

Prior to randomization, the selected adolescents will complete a self-administered baseline questionnaire to collect their sociodemographic data and measure symptoms of different mental health disorders.

The following 8 instruments will be used: the Family adaptability, partnership, growth, affection, and resolve; the Self-Reporting Questionnaire to assess mental health problems; the 8-item Patient Health Questionnaire depression scale, to assess depression symptoms; the Generalized Anxiety Disorder 7-item scale to assess anxiety symptoms; the PTSD Checklist to measure symptoms; the 25-item Connor Davidson Resilience Scale to measure resilience; the Rosenberg scale to measure self-esteem; and the Manchester Short Assessment of Quality of Life to measure quality of life.

These will be administered by research assistants who are blinded to the arm allocation and will be repeated at the end of the follow-up.

The experimental intervention will be carried out using DIALOG+, for which each teacher will be provided a tablet with the application and will undertake a 90-minute standardized training session on its use. DIALOG+ consists of 2 main parts: (1) a person-centered assessment, whereby the teachers invite the students to rate their satisfaction with different life domains, followed by (2) a 4-step approach based on the principles of solution-focused therapy [34]. Following review of the scores across the DIALOG scale, which includes comparing the current ratings with the ratings obtained from any previous session, up to 3 of the areas that are listed on the DIALOG scale are chosen to be discussed in more detail. The 4-step solution-focused approach is used to structure the discussion to identify patients’ resources and develop solutions to deal with the adolescents’ concerns. At all times, the ratings on the scale are referred to in order to underpin and contextualize the discussion. Step 1, *Understanding*, elicits contextual information about the area under discussion and establishes what is working in that area. Step 2, *Looking Forward*, asks the adolescent to adopt a future perspective and think about the “best-case scenario” within that domain as well as the smallest improvement that can be made to incrementally move up the rating scale. Step 3, *Considering Options*, invites the adolescent to reflect on what he/she and others can do to improve their quality of life. Finally, step 4, *Agreeing on Actions*, summarizes the discussion, and a list of actions is generated and input into the application. Ultimately, the teacher and student together will develop an action plan comprising individual action items for each of the discussed areas to be completed before the next session.

The control arm’s meetings will be conducted in the usual manner as teachers conduct counseling. Considering that most teachers have no training in the use of psychological techniques, it is not expected that they will be used.

During this phase, teachers who were assigned to the intervention group will record voice notes expressing their thoughts and opinions about the implementation process using the think-aloud method.

We will assess process measures to better understand the feasibility of the intervention for application in schools in Colombia. We will assess teacher and student recruitment and retention when reporting reasons for refusal and reasons for loss to follow-up, as well as the number and frequency of sessions conducted in both arms. Feasibility criteria will include recruitment of at least 85% of anticipated participants and a retention rate of at least 75%. We will also have at least two-thirds of sessions completed as planned (on average at least 4 sessions) as an additional feasibility criterion.

#### Consolidation

Finally, we will conduct 2 focus groups with teachers and students. Both groups will be asked to discuss their experience, perceptions, and possible changes or modifications required to adapt DIALOG+ to the school setting. Adolescents will be asked if they perceived changes in their mental well-being. The data obtained will help assess the viability of using DIALOG+ in a school context in vulnerable populations in postconflict areas in Colombia.

### Participants

This study will take place in one PDET the Chaparral municipality of Tolima. The recruitment of school teachers or counselors and adolescents will take place in 2 public schools through invitation. Each teacher will invite 5 adolescents whom they consider to be in need of counseling or additional support. Participants will be excluded from the study if they intend to change towns or schools in the near future or if they cannot participate for the full duration of the study.

All adolescents and their parents or guardians and teachers will be asked to provide assent and informed consent by signing an assent and informed consent form before any data collection begins. During the consent process, researchers will ensure that participants and parents or guardians are aware of their right to decline participation at any stage of the study and that withdrawing participation will not affect their rights or access to support.

Participants who withdraw from the study will be able to ask for their data to be removed from analysis. If a participant wishes to withdraw from the study, researchers will record the date of withdrawal and reasons for withdrawal (if provided).

### Sample Size

Consistent with the exploratory study design, convenience sampling will be performed. We will conduct the study with 14 teachers and 70 students. The population of adolescents attending the 2 participating schools is 1494. However, the number of participating teachers is limited both by the number of teachers in each school and by the capacity of the teachers to conduct counseling. In each school, there is only one school counselor who is a psychology professional. The teachers who conduct counseling do so because they are interested in the well-being of their students. This is an exploratory study to determine whether it is feasible and acceptable to adapt DIALOG+ in the school setting, with quantitative outcomes to provide estimates of effects that could be used to design a larger study if our findings are supportive of this. Since this is an exploratory trial, the power to detect meaningful differences with statistical significance will be limited. With an intraclass correlation coefficient of 0.05, which we estimated from other studies we have conducted, the planned sample size will allow us to detect an effect size of 0.80 with 78% power and a smaller effect size of 0.5 with 40% power. In the case of the Manchester Short Assessment of Quality of Life, usually the primary outcome in DIALOG+ studies, an effect size of 0.80, would be equivalent to a change of approximately 6 points in total (eg, 1 point on a 7-point scale each on 6 out of 12 items, or 3 points each on 2 areas of life), which reflects a meaningful change.

### Ethical Considerations

This protocol has been approved by the Pontificia Universidad Javeriana Faculty of Medicine Institutional Research and Ethics Committee (CIEI-0239-21) and the Queen Mary University of London Research Ethics Committee (ref QMERC20.226). The study was prospectively registered on the ISRCTN registry (ISRCTN14396374). Written assent from the adolescents with consent from the parents or guardians and written consent from the teachers will be obtained prior to any study procedures being conducted.

### Measures

Data will be collected using a standardized paper-based case report form. At baseline and after the intervention phase of follow-up, we will collect sociodemographic data from both teachers and students and the previously mentioned mental health measures.

### Data Management and Analysis

This study will use a mixed methods design. Quantitative and qualitative aspects will be triangulated in the overall evaluation of the intervention. Both qualitative and quantitative results will be presented for a global analysis of the intervention. For variables measured at 2 time points (baseline and 6-month follow-up), we will report measures of central tendency and dispersion in accordance with the data distribution, and significance tests will be performed. The analysis will use intention-to-treat analysis by including all students in the arm to which they were randomized, whether or not they received the intervention, and including all students in the analysis by using multiple imputation where outcomes are missing. To generate parameter estimates for any changes in outcomes for students using DIALOG+, mean differences (with standard deviations) and effect sizes over 2 time points (baseline and after the intervention period) will be calculated.

Qualitative data will be analyzed using thematic content analysis techniques [37]. Qualitative data will be analyzed using a framework of theoretical domains of behavioral change to understand the experience and issues concerning behavioral change that could be related to the acceptability and use of DIALOG+, as well as possibilities for improving implementation. This will serve to develop concrete plans for the implementation and evaluation of the strategy, such as clinical trials that evaluate the effectiveness of the tool with a larger number of participants. All interviews will be transcribed and analyzed using NVivo software [38], which creates graphical displays and facilitates thematic content analysis.

### Dissemination

We will disseminate the knowledge generated to academic audiences through peer-reviewed publications in high-impact scientific journals in Spanish and English and international conference presentations. We will circulate the publications and presentations via the extensive networks of the investigators. Dissemination events will be conducted to ensure that the results are fed back to the Secretary of Education and to the principals of participating schools.

## Results

Study recruitment was completed in March 2022, and follow-up is anticipated to last through November 2022. Each phase is expected to produce data that will facilitate the use of the DIALOG+ intervention for Colombian adolescents. The adaptation phase is expected to provide insight into the modifications that the digital intervention requires in terms of domains discussed and the interface. The exploration phase (the longest phase) is expected to provide the most information regarding the feasibility and acceptability of implementation. Baseline and follow-up questionnaires will provide evidence regarding acceptability and parameter estimates for the impact of the intervention. Lastly, the consolidation phase will report on the experiences and perceptions of the experience for both educators and adolescents.

## Discussion

The increasing prevalence of mental health disorders in adolescents has highlighted the importance of addressing their mental health needs, particularly in contexts where they have experienced violence and armed conflict and especially after the COVID-19 pandemic. To meet these needs, an innovative approach is required where discussion regarding mental health cannot be limited only to a clinical and health care setting. Increasing the role that school settings have in mental health promotion may improve early identification and intervention with adolescents who are struggling [39]. This approach can also provide support to health security systems that face a challenge in bridging the gap and inequities in health care access such as the one in Colombia.

With this study, through different phases, we aim to assess the feasibility of implementing DIALOG+ in the school setting of adolescents in vulnerable areas in Colombia.

Studies on school-based interventions developed to increase adolescent well-being suggest that interventions may be successfully delivered by teachers and can improve outcomes by reducing symptomatology and encouraging early referral [39-41]. Studies on such interventions in adolescents exposed to conflict are scarce. Experiences with Palestinian adolescents of the Gaza strip revealed that school counseling sessions had a positive impact on PTSD symptomatology; however, no other mental health disorders were evaluated [42]. To the best of our knowledge, the adaptation and assessment of DIALOG+ as a resource-oriented and cost-effective intervention for school adolescents in postconflict Colombia provides an innovative approach in both the school and a postconflict context, and this tool is the first of its kind.

Studies on implementing DIALOG+ have been conducted in adults but no efforts have been undertaken to support the mental health skill development of teaching and counseling staff within the education system. The school setting is an area in which contact with children and adolescents with mental health problems is frequent and ongoing. Therefore, this is a critical and new area of research, as support from school staff is likely to be the only help that those children and adolescents will receive in this setting. Building capacities in teachers and school counselors through mental health courses would be complemented extraordinarily with a study that shows the effectiveness of DIALOG+ as a tool to be used by teachers and counselors in the school environment. This study proposes to evaluate feasibility and acceptability as a first step toward wider testing and possible implementation. It is important to highlight that the content and design of this study were developed through workshops, consultations, and discussions among the researchers, and considerations from local stakeholders with regard to the local context, health care priorities, and logistics of conducting this intervention were acknowledged.

The study design (an exploratory randomized controlled trial) may provide limited conclusions on the effectiveness of DIALOG+ owing to its small sample sizes, which provides limited statistical power and generalizability, but will still provide parameter estimates that will be essential for larger trials should these be indicated. However, we believe it will provide valuable insight into the feasibility and acceptability of DIALOG+ in the school environment, which is the primary aim of the study.

In conclusion, the results obtained in this study will provide valuable information regarding the feasibility of using a digital intervention such as DIALOG+ in the school setting to improve the well-being of vulnerable adolescents in Colombia. Experience obtained will deliver evidence that can be useful in other conflict-affected areas of the world and can provide insight into the usefulness of school-based interventions in general.

## Abbreviations

PDET: Development Program with a Territorial Approach
PTSD: posttraumatic stress disorder
